# PARK7 Catalyzes Stereospecific Detoxification of Methylglyoxal Consistent with Glyoxalase and Not Deglycase Function

**DOI:** 10.1021/acs.biochem.3c00325

**Published:** 2023-10-26

**Authors:** John S. Coukos, Chris W. Lee, Kavya S. Pillai, Hardik Shah, Raymond E. Moellering

**Affiliations:** † Department of Chemistry, The University of Chicago, 929 E. 57th Street, Chicago, Illinois 60637, United States; ‡ University of Chicago Medicine Comprehensive Cancer Center Metabolomics Platform, The University of Chicago, 900 E. 57th Street, Chicago, Illinois 60637, United States

## Abstract

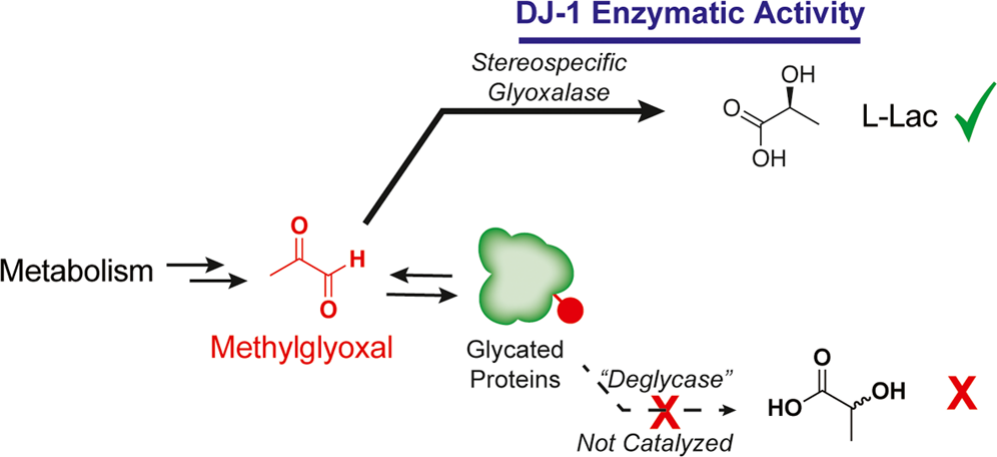

The protein PARK7 (also known as DJ-1) has been implicated in several diseases, with the most notable being Parkinson’s disease. While several molecular and cellular roles have been ascribed to DJ-1, there is no real consensus on what its true cellular functions are and how the loss of DJ-1 function may contribute to the pathogenesis of Parkinson’s disease. Recent reports have implicated DJ-1 in the detoxification of several reactive metabolites that are produced during glycolytic metabolism, with the most notable being the α-oxoaldehyde species methylglyoxal. While it is generally agreed that DJ-1 is able to metabolize methylglyoxal to lactate, the mechanism by which it does so is hotly debated with potential implications for cellular function. In this work, we provide definitive evidence that recombinant DJ-1 produced in human cells prevents the stable glycation of other proteins through the conversion of methylglyoxal or a related alkynyl dicarbonyl probe to their corresponding α-hydroxy carboxylic acid products. This protective action of DJ-1 does not require a physical interaction with a target protein, providing direct evidence for a glutathione-free glyoxalase and not a deglycase mechanism of methylglyoxal detoxification. Stereospecific liquid chromatography–mass spectrometry (LC-MS) measurements further uncovered the existence of nonenzymatic production of racemic lactate from MGO under physiological buffer conditions, whereas incubation with DJ-1 predominantly produces l-lactate. Collectively, these studies provide direct support for the stereospecific conversion of MGO to l-lactate by DJ-1 in solution with negligible or no contribution of direct protein deglycation.

## Introduction

Protein DJ-1, produced by the *PARK7* gene, has been implicated in familial Parkinson’s disease, where homozygous or compound heterozygous mutations have been shown to lead to early onset of disease.^1−4^ DJ-1 is a 189-amino acid protein that is ubiquitously expressed and is primarily cytosolic,^5^ although it is reported to be present in other cellular compartments including the mitochondria and nucleus.^6,7^ In addition to its causative role in Parkinson’s disease, DJ-1 appears to have relevance in a variety of diseases and other physiological contexts. DJ-1 has also been implicated as a potential oncogene in breast and other cancers.^8−10^ Mice with DJ-1 deficiency have been shown to develop glucose intolerance and reduction in β-cell area with age,^11^ suggesting that DJ-1 might provide a protective function in the context of diabetes. This idea is bolstered by experiments that show that DJ-1 is highly upregulated by islet cells in response to glucose challenge.^12^ Additionally, DJ-1 may preserve proper cellular function with aging as exemplified by studies that show DJ-1 is integral to maintenance of regulatory T cell function in aged mice.^13^ In addition to the complex roles that DJ-1 plays in pathophysiology, many distinct molecular and cellular functions have been ascribed to DJ-1 including chaperone function, transcriptional regulation, protection against oxidative stress, and maintenance of mitochondrial function.^14−17^ More recently, reports have highlighted the role that DJ-1 plays in detoxification of reactive metabolites produced during glycolysis such as α-oxoaldehyde methylglyoxal^18−21^ and 1,3-bisphosphoglycerate (1,3-BPG), a highly reactive metabolite that can acylate a variety of nucleophilic amines in cellular proteins and metabolites.^22−24^

Methylglyoxal (MGO) is a highly reactive metabolite produced by the degradation of the triose phosphates glyceraldehyde phosphate (GAP) and dihydroxyacetone phosphate (DHAP), which interconvert within glycolysis through the enzyme triosephosphate isomerase. MGO can chemically modify a variety of nucleophilic biomolecules including proteins, nucleic acids, and even metabolites.^25−31^ The abundance of MGO-modified proteins has been associated with a number of diseases including diabetes, cancer, neurodegeneration, and aging,^32−35^ with Parkinson’s disease-specific connections potentially being explained by a role for DJ-1 in protecting against accumulation of glycated α-synuclein.^36,37^ MGO is principally detoxified by the glyoxalase system, which consists of the enzymes GLO1 and GLO2.^38−40^ MGO reacts with reduced glutathione (GSH) to form a reversible hemithioacetal, which can be converted to lactoyl glutathione (Lac-GSH) by Zn^2+^ metalloenzyme GLO1.^41^ GLO2 then may hydrolyze Lac-GSH to recycle the GSH and produce d-lactate.^42^d-Lactate may be further metabolized to pyruvate by lactate dehydrogenase D (LDHD).^43^

DJ-1 was first reported to detoxify MGO and related compounds in a GSH-independent manner in 2012.^18^ Subsequent reports have highlighted C106 and H126 as critical active site residues that mediate this function as a catalytic dyad akin to those found in cysteine proteases such as the peptidase C56 family with which DJ-1 shares sequence homology.^18,44^ As with GLO1/GLO2, the detoxification of MGO by DJ-1 produces lactate. Despite the fact that a number of studies have examined the role of DJ-1 in the detoxification of MGO, the exact mechanism by which this occurs is still hotly debated. While initial studies seemed to indicate that DJ-1 directly catalyzes the conversion of MGO to lactate in solution,^18^ several recent studies have advanced the idea that DJ-1 acts as a so-called deglycase, i.e., an enzyme that removes reversible MGO modifications directly from proteins or nucleic acids to produce lactate,^10,36,45−47^ prompting a debate in the literature.^48−53^ The specific mechanism by which DJ-1 detoxifies MGO could have significant implications for the scope of protection against glycative stress that DJ-1 affords and, by extension, the cellular function of DJ-1 and its role in disease. Thus, we sought to investigate the precise role that DJ-1 plays in the detoxification of MGO and protection of proteins from glycation.

## Results and Discussion

**Figure 1:**
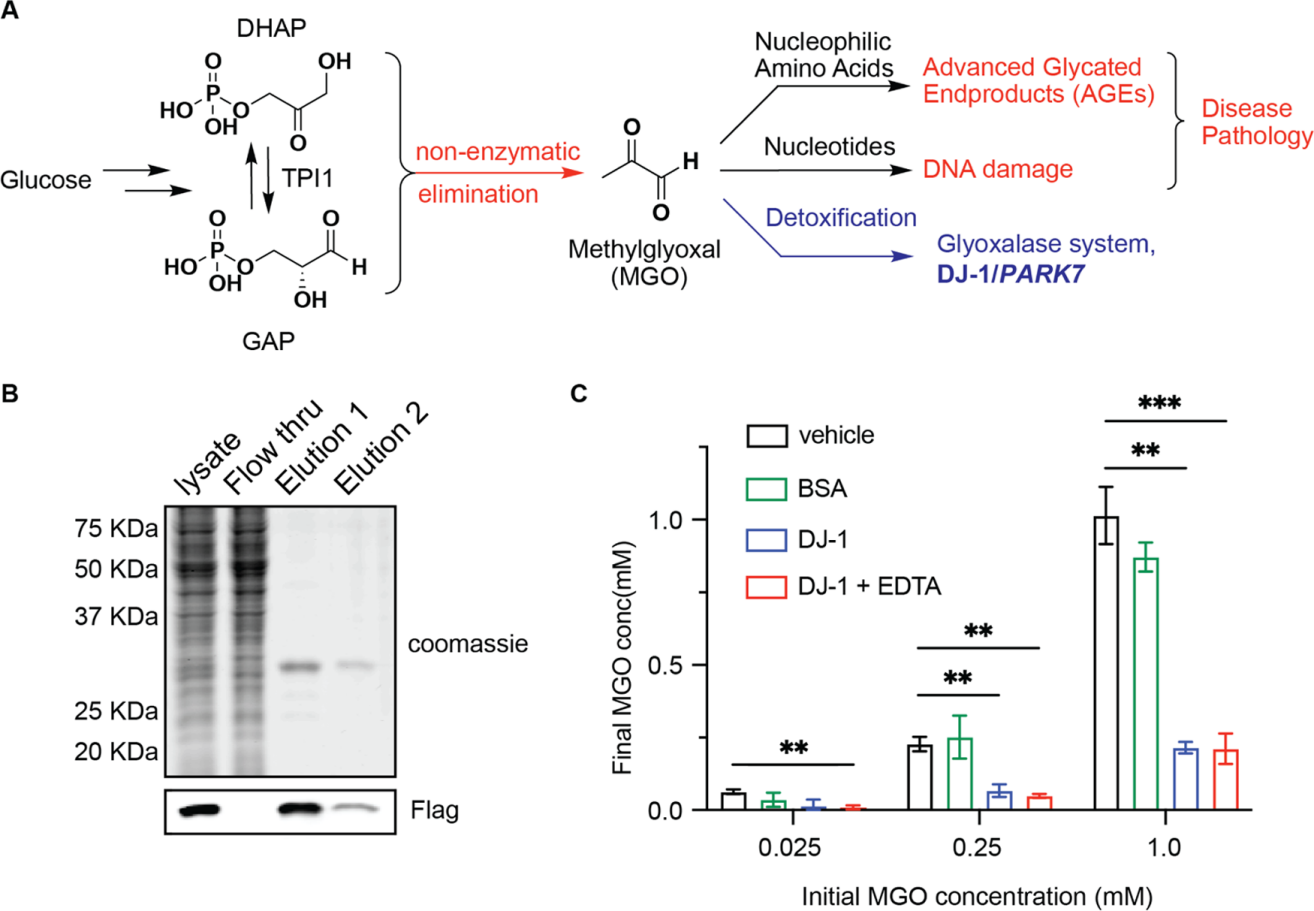
Purified DJ-1 actively detoxifies methylglyoxal. (A) Schematic depicting the formation and cellular fates of methylglyoxal. (B) Representative Coomassie gel and anti-FLAG Western blot of purified FLAG-DJ-1 isolated from HEK293T cells stably overexpressing Flag-DJ-1. (C) MGO quantification in recombinant assays containing the indicated MGO concentration and protein condition following incubation for 24 h at 37 °C. Data plotted in (C) are mean ± SEM from *n* = 6 independent biological replicates. Statistical analyses are by ordinary one-way analysis of variance (ANOVA). *p < 0.05; **p < 0.01; ***p < 0.001; and ****p < 0.0001.

Accumulation of MGO-derived adducts on proteins, collectively known as advanced glycation end products (AGEs), has been implicated as correlated and causal in a variety of diseases (Figure 1A). Multiple routes of detoxification of MGO have been identified, most notably the glutathione-dependent conversion to d-lactate by the combined action of GLO1 and GLO2 (collectively referred to as the glyoxalase pathway in mammals). More recently, Parkinson’s disease-associated protein DJ-1 has been implicated in detoxification of MGO, although the mechanism by which it does so is still a matter of debate.^48^ To enable the study of this mechanism, we stably expressed recombinant DJ-1 with a C-terminal FLAG-HA tag in HEK293T cells, which were used for anti-FLAG purification of the active enzyme (Figure 1B). The structure of the recombinant DJ-1 was confirmed by proteomic analysis and quantification of protein concentration performed using gel-based analysis (Figure S1A–C and Table S1). We confirmed the activity of purified DJ-1 in MGO metabolism assays across several conditions and dose ranges (Figure 1C). Incubation of purified DJ-1 with MGO showed a significant decrease in MGO levels across a range of μM–mM concentrations (Figure 1C), which contrasted with the negligible MGO depletion observed when incubated with the control BSA carrier protein alone to account for the potential removal of free MGO by adduct formation. Furthermore, addition of EDTA to the reaction did not significantly affect the observed detoxification of MGO by DJ-1, confirming that the activity detected was not from low-level GLO1 or alternative metalloenzyme contamination.

While previous work has established the role of DJ-1 in detoxifying MGO and protecting proteins from glycation, significant debate still remains about the mechanism by which this is accomplished.^48^ Studies have suggested that DJ-1 might behave as a glutathione-independent glyoxalase,^18,50,51^ a glutathione-dependent glyoxalase,^54^ or even an adduct-directed deglycase enzyme capable of removing MGO-derived modifications from proteins and nucleotides directly.^19,20^ While several recent papers have bolstered support for a glutathione-free glyoxalase model over a deglycase model, these papers either addressed proposed substrates that were not the early glycation hemiaminals and hemithioacetals^51,52^ that were proposed in a deglycase model or relied on mechanistic inference from kinetic assays that were indirect and potentially open to interpretation.^53^ Additionally, there are still conflicting reports regarding stereochemistry of the lactate product of DJ-1 without a satisfactory rationale to resolve these disparate results.^19,51,54^ To conclusively rule out a deglycase model, it was necessary to determine whether a direct interaction with target proteins is required to protect them from glycation. To do so, we utilized an analogue of MGO with an alkyne handle for click chemistry derivatization to detect and quantify MGO-derived protein modifications on purified proteins, cell lysates, and/or live cell proteomes. We devised a modified synthetic route for this α-oxoaldehyde, terminal alkyne-containing probe (MG-alkyne), based on previously reported synthetic routes.^55,56^ Our route consisted of three steps to generate a stable acetal-protected precursor, which could undergo facile deprotection (Figure S2A) and direct use in biological experiments; this is necessary due to the reactive and unstable nature of the α-oxoaldehyde moiety. Once neutralized, the concentration of the probe could be determined by derivatization with amino guanidine and quantification based on a calibration curve generated by serial dilutions of 3-amino-1,2,4-triazine (Figure S2B), a protocol originally developed to similarly quantify the MGO concentration.^57^

**Figure 2:**
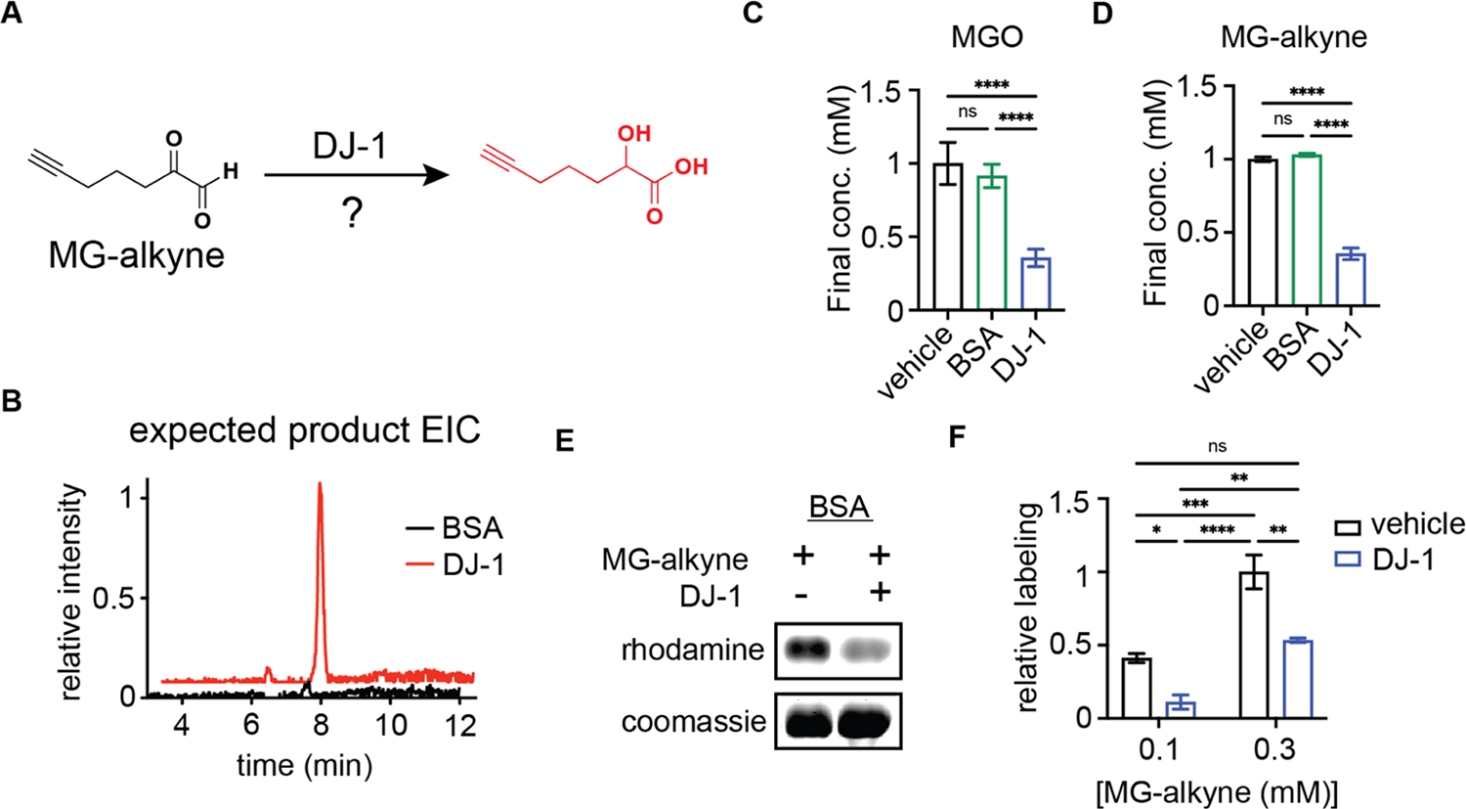
DJ-1 detoxifies an alkynylated analogue of MGO and protects proteins from modification. (A, B) Structure (A) and extracted ion chromatogram (B) of the predicted lactoyl-alkyne metabolic product of MG-alkyne treated with equal amounts of DJ-1 or BSA for 24 h at 37 °C. (C) Quantification of remaining MGO from the reaction where 1 mM MGO was treated with equal amounts of recombinant DJ-1 or BSA or with the vehicle for 24 h at 37 °C. (D) Quantification of remaining MG-alkyne from the reaction where 1 mM MG-alkyne was treated with equal amounts of DJ-1 or BSA or with the vehicle for 24 h at 37 °C. (E) Representative rhodamine gel of 2 mg/mL BSA treated with 300 μM MG-alkyne as well as recombinant DJ-1 or vehicle for 24 h at 37 °C. (F) Quantification of labeling of 2 mg/mL BSA treated with 100 or 300 μM MG-alkyne as well as recombinant DJ-1 or vehicle for 24 h at 37 °C. Data plotted in (C–F) are mean ± SEM from *n* = 6 (C), 4 (D), or 3 (F) independent biological replicates. Statistical analyses are by ordinary one-way analysis of variance (ANOVA). *p < 0.05; **p < 0.01; ***p < 0.001; and ****p < 0.0001.

We first confirmed that treatment of cell lysate with MG-alkyne followed by click chemistry derivatization with rhodamine azide and in-gel fluorescence scanning revealed dose-dependent labeling of the native HeLa proteome by the MG-alkyne probe; this labeling is due to the formation of various stable adducts on target proteins (Figure S2C). Since the MG-alkyne probe is a reactive proxy for MGO, we sought to determine whether DJ-1 could enzymatically detoxify this larger substrate and whether this activity could prevent or rescue MGO-mediated protein adduct formation. We treated MG-alkyne with purified DJ-1 or with BSA carrier protein and then performed targeted LC-MS analysis on the resultant reactions. Because MGO is metabolized to lactate by DJ-1, we hypothesized that the solution glyoxalase activity of DJ-1 could likewise convert MG-alkyne into the corresponding lactoyl-alkyne product (Figure 2A). As expected, MG-alkyne levels were reduced and the corresponding lactoyl-alkyne product was generated in DJ-1-containing reactions but not the BSA control (Figure 2B).

Treatment of an equal concentration of MGO or MG-alkyne with either DJ-1 or BSA control showed comparable degrees of turnover with both substrates by DJ-1, suggesting that despite the larger alkynyl tail, DJ-1 recognizes and catalyzes the conversion of the dicarbonyl reactive headgroup into the corresponding α-hydroxyl carboxylate (Figure 2C,D)—an activity that would be somewhat unexpected if DJ-1 were acting on diverse protein adducts with the tail group facing toward the solvent. Finally, we incubated BSA, which is readily modified by MG-alkyne (Figure S2C), with different concentrations of MG-alkyne for 24 h in the presence or absence of DJ-1. BSA reactions containing DJ-1 showed significantly less labeling by MG-alkyne relative to control reactions even at this extended incubation period, confirming that the MG-alkyne glyoxalase activity by DJ-1 can functionally protect proteins from stable modification (Figure 2E,F).

**Figure 3:**
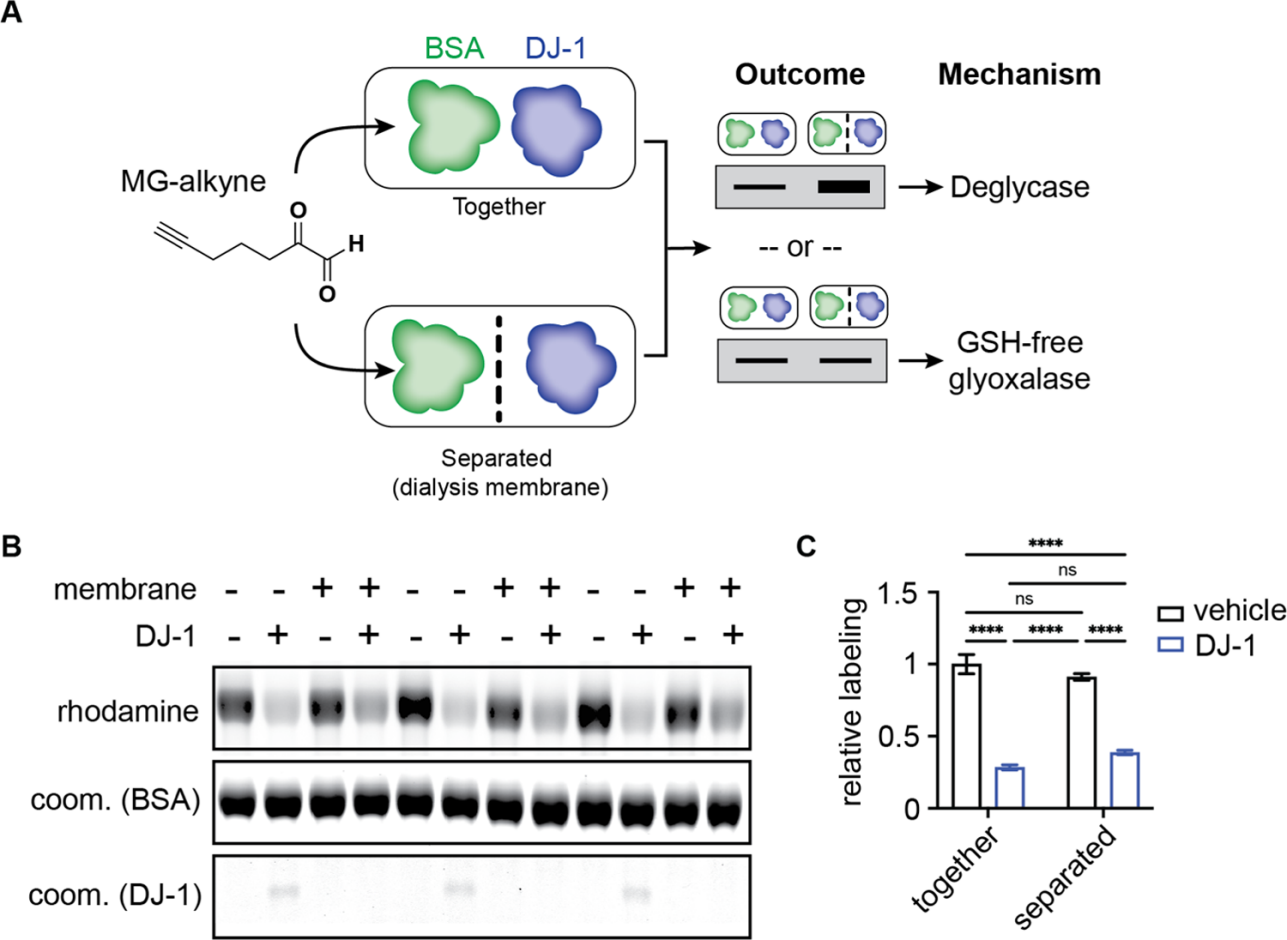
DJ-1 protects proteins from glycation with or without a physical interaction with target proteins. (A) Schematic depicting experiments where BSA and recombinant DJ-1 are incubated with MG-alkyne together or separated by a dialysis membrane alongside predicted interpretations. (B, C) Rhodamine gel (B) and quantification of labeling (C) of BSA incubated with 100 μM MG-alkyne in the presence or absence of recombinant DJ-1 with and without separation by a dialysis membrane. Data plotted in (C) are mean ± SEM from *n* = 3 independent biological replicates. Statistical analyses are by ordinary one-way analysis of variance (ANOVA). ****p < 0.0001.

While our experiments here cumulatively support an in-solution glyoxalase activity for DJ-1, the DJ-1-mediated detoxification of MGO and MG-alkyne could in theory result from the direct removal of transient dicarbonyl modifications (e.g., hemiaminals or similar) on target proteins (BSA) or DJ-1 *in trans*, as argued in some previous studies.^19^ Because it is challenging to directly uncouple these competing mechanisms, we sought to detect and quantify MG-alkyne modifications on a target protein when DJ-1 is physically separated from the said target protein population. Specifically, we developed an assay where BSA was incubated with MG-alkyne and either coincubated with DJ-1 in the same compartment or physically separated from DJ-1 by a dialysis membrane (Figure 3A). This separation would prevent the DJ-1 protein molecules from physically interacting with the BSA protein molecules, as would be necessary for a direct removal of dicarbonyl adducts in a deglycase mechanism, but would allow the free diffusion of MG-alkyne between compartments. As in previous experiments, we observed significant MG-alkyne modification of BSA in this assay. Importantly, we found that the samples where BSA and DJ-1 were separated by a dialysis membrane and the samples where they were coincubated showed no appreciable difference in the degree of modification by the MG-alkyne probe (Figure 3B,C). These data confirm that the physical interaction between DJ-1 and dicarbonyl modified protein targets is not necessary for the observed detoxification and provides direct evidence for the in-solution glyoxalase activity for MGO conversion to lactate and protection against protein glycation.

**Figure 4:**
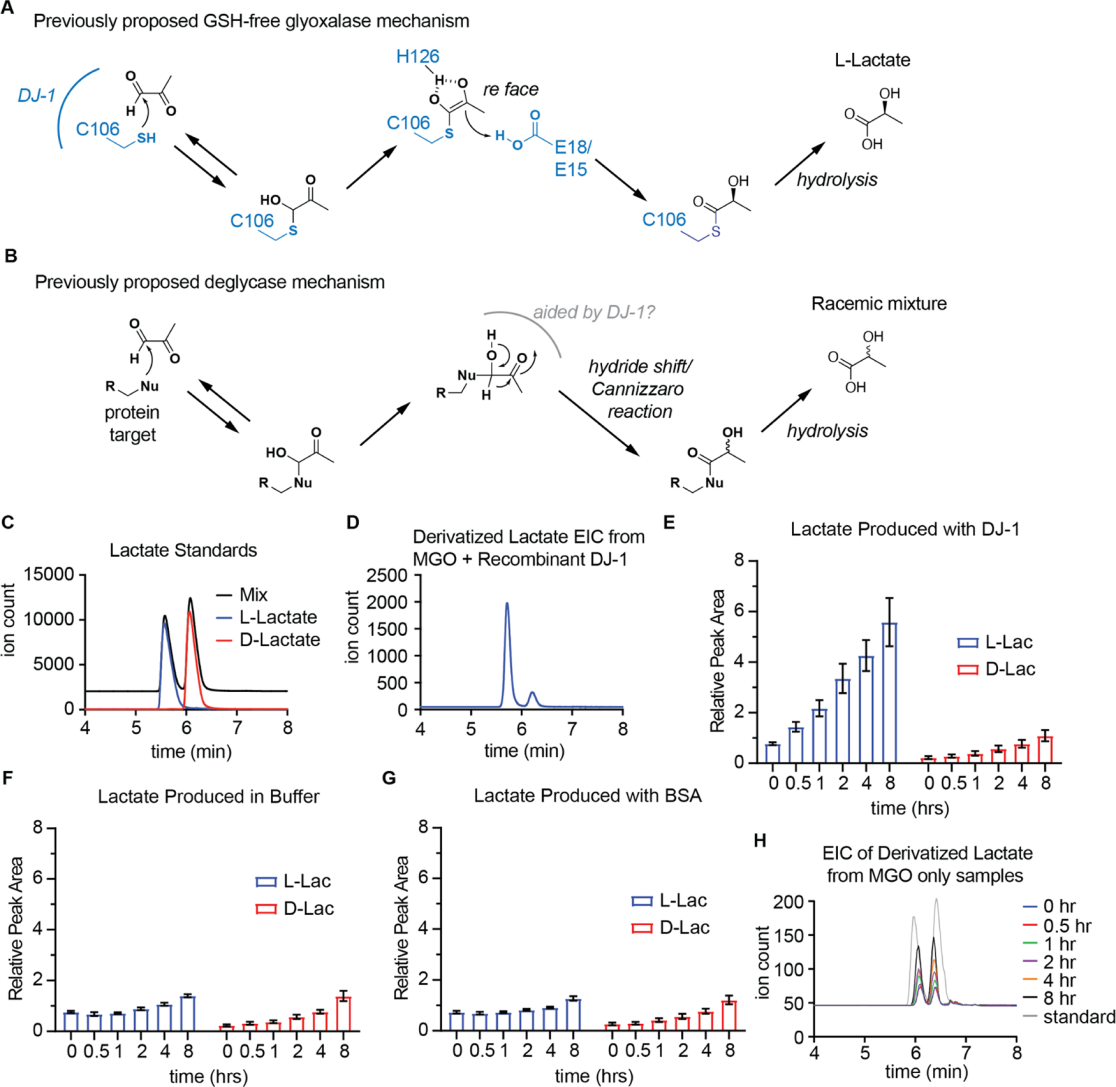
Lactate product stereochemistry is consistent with a GSH-free glyoxalase mechanism. (A, B) Previously proposed DJ-1 glyoxalase (A) and deglycase (B) mechanisms and predicted product lactate stereochemistry. (C, D) LC-MS separation of DATAN-derivatized lactate standards (C) and DJ-1-catalyzed products (D). Both chromatograms show the extracted ion chromatogram (EIC) for the DATAN-derivatized product. (E–G) Quantification of the integrated peak area of l- and d-lactate formed by DJ-1, BSA, or PBS treated with MGO for 0–8 h at 37 °C. (H) Representative chromatograms of derivatized l- and d-lactate formed from PBS treated with 1 mM MGO for 0–8 h at 37 °C along with l- and d- lactate synthetic standards. Data plotted in (E–G) are mean ± SEM from *n* = 4 independent biological replicates normalized across all conditions.

The proposed mechanism for a glutathione-free glyoxalase mechanism predicts that DJ-1 would catalyze the enantioselective formation of l-lactate (Figure 4A), whereas putative deglycase activity is expected to produce a racemic mixture of l- and d-lactate (Figure 4B);^19,48^ the fact that direct deglycation of protein adducts would also infer a heterogeneous substrate pool mediated by diverse protein surfaces would also strongly support a mixture of lactate stereoisomers being produced under this mechanism. Despite these mechanistic rationalizations, some previous studies have indeed observed mixtures of d- and l-lactate generated by DJ-1,^19,54^ while others show significant preference for the l-lactate isomer,^51,58^ suggesting that both glyoxalase and deglycase mechanisms could be operating in parallel. To help distinguish between these two potential mechanisms and clarify the disparity in findings between previous studies with respect to stereochemistry of the lactate product, we utilized a chemical derivatization method to allow separation and quantification of the l- and d-enantiomers of lactate.^59^ Using synthetic standards, we validated the ability to achieve baseline separation for accurate quantification of the diastereomeric derivatives of the lactate enantiomers (Figure 4C). Utilizing this approach, we performed kinetic LC-MS quantification of the measurement of the l- and d-lactate produced by incubating recombinant DJ-1 with MGO, which revealed that the vast majority, about 83%, of the lactate present was the l-enantiomer (Figure 4D). This was inconsistent with either of the two proposed mechanisms operating exclusively and left open the possibility that both activities could be present.

**Figure 5:**
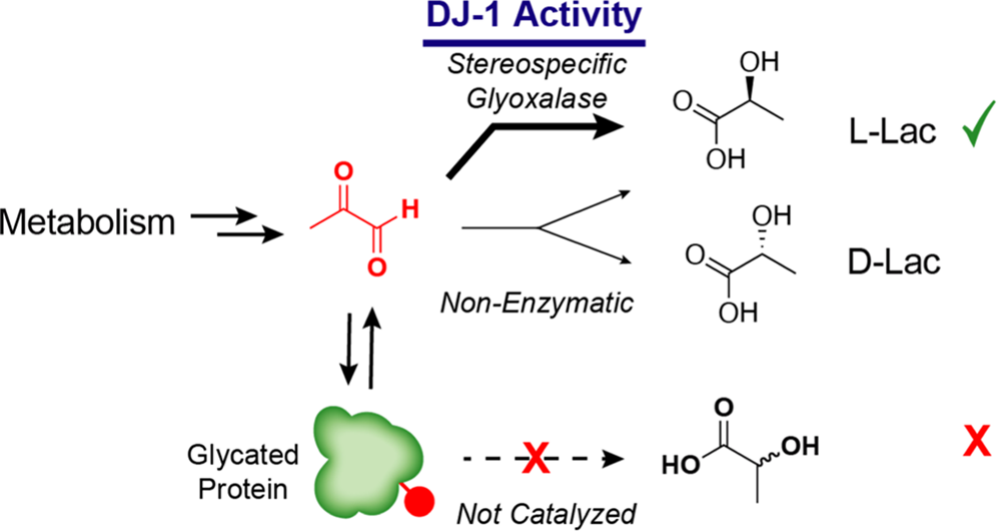
Schematic depicting the stereospecific glyoxalase activity of DJ-1 supported by this study.

To gain further insights into the production of l- and d-lactate by DJ-1, diastereomeric derivatization was again utilized to look at the kinetics of lactate production in an enantiospecific manner. Time series incubations over an 8 h time period were performed where MGO was incubated with either DJ-1, equal concentration of BSA carrier protein, or PBS buffer alone, and the relative rates of l- and d-lactate production were quantified (Figure 4E). Unexpectedly, although the samples incubated with DJ-1 showed a much higher rate of overall lactate production, all three conditions showed some time-dependent production of lactate (Figure 4E–G). DJ-1-catalyzed reactions produced markedly more l-lactate than that of BSA or PBS control reactions. By contrast, the production of d-lactate was identical across DJ-1, BSA, and PBS samples. This is consistent with a model where l-lactate is produced enzymatically by DJ-1, but a racemic mixture of l-lactate and d-lactate is arising from nonenzymatic, spontaneous conversion of MGO to lactate in buffer alone. Indeed, the amounts of l- and d-lactate produced in reactions without DJ-1 were identical (Figure 4H). To confirm that the observed nonenzymatic production of lactate from MGO is not caused by our derivatization procedure, we incubated MGO alone in PBS and directly quantified total lactate over 8 h by targeted LC-MS/MS. These experiments confirmed the time-dependent production of lactate from MGO without the presence of an enzymatic catalyst and in the absence of derivatization (Figure S3A,B). To rule out lactate production from a biological contaminant, we compared production of lactate from MGO in PBS that had been autoclaved immediately prior to the experiment, which showed no difference in lactate production (Figure S3). To assess factors that regulate the nonenzymatic conversion, we incubated MGO in PBS or deionized water at either 4 or 37 °C. There was markedly reduced production in conditions where the MGO was incubated in water or at 4 °C, suggesting that temperature and buffer composition play a role in the rate of nonenzymatic conversion of MGO to lactate. Together, these data thus support the exclusive enzymatic production of l-lactate by DJ-1, which is confounded by the background production of equimolar d- and l-lactate in solution by as of yet unknown mechanisms. This observation, which has not been previously reported to the best of our knowledge, provides a parsimonious explanation for the previously observed production of d- and l-isomers in enzymatic reactions and combined with our biochemical studies here strongly supports a stereospecific glyoxalase activity for DJ-1 to form l-lactate (Figure 5). It additionally suggests that the variable ratios of l- and d-lactate observed in previously published work with DJ-1 could be caused, at least in part, by variable ratios of enzymatic versus nonenzymatic production of lactate, likely dependent on the amount and degree of activity of DJ-1 used as well as the particular reaction conditions.

## Discussion

This study sought to determine the mechanism by which DJ-1 detoxifies the reactive metabolite methylglyoxal (MGO). This study confirmed the role that DJ-1 plays in the detoxification of MGO as well as the enantioselective production of l-lactate by DJ-1 is consistent with a glutathione-free glyoxalase mechanism. We showed that DJ-1 protects proteins from glycation by MGO and an alkyne-containing analogue without the need to physically interact with target proteins, suggesting that the protection stems from direct detoxification of dicarbonyl species in solution rather than the removal of MGO from modified proteins. While these experiments utilize distinct probes and experimental setups to interrogate unique MGO-mediated modifications from previous studies including Gao et al.,^51^ they collectively support the same conclusion that a direct interaction with DJ-1 is not required to protect a target biomolecule from reversible or stable MGO modification. Additionally, this study demonstrates for the first time that MGO can nonenzymatically convert to lactate under physiologically relevant conditions and time courses. This unavoidable production of racemic lactate by MGO is likely to be both physiologically and experimentally important. In particular, this source of lactate production must be accounted for in any assays that seek to study the enzymatic activity and parameters of proteins such as DJ-1 and GLO1, which are involved in MGO detoxification. Additionally, the finding may help explain the variable reports of the nature of lactate enantiomers produced by DJ-1, which have primarily been characterized by inference from enzymatic assays and not measured directly.^48^ From a biological perspective, nonenzymatic detoxification of MGO may help explain why cells can tolerate loss of methylglyoxal detoxifying enzymes under unstressed conditions.^60^ Most importantly, our enzymatic assays conclude that DJ-1 activity results in almost exclusive production of l-lactate, which is consistent with a stereospecific glyoxalase mechanism and not a deglycase mechanism for DJ-1-catalyzed lactate production (Figure 5). Collectively, we posit that these experiments offer the most direct evidence in support of this mechanism and adds to recent studies^51,58^ and the overall body of evidence clarifying the role of DJ-1 in MGO detoxification.

Beyond activity on MGO, DJ-1 has recently been shown to act on other reactive metabolites such as a putative cyclized product of 1,3-BPG.^24^ Connecting the role of DJ-1 in the detoxification of such metabolites with its various reported cellular functions may provide insights into why the mutation of DJ-1 is causative in familial Parkinson’s disease. Moreover, future studies should focus on identifying important cellular targets of these metabolites and understanding how DJ-1 may modulate the function of these proteins through the regulation of glycative stress.

## Supporting Information Available

DJ-1-derived tryptic peptides detected by proteomic analysis (XLSX)
Quantification of DJ-1 for enzymatic assays, activation and evaluation of the MG-alkyne probe, metabolomic characterization of nonenzymatic lactate production, and materials/experimental details (PDF)

## Accession Codes

PARK7/DJ-1: Q99497

## Abbreviations

MGO: methylglyoxal
GLO1: glyoxalase 1
GLO2: glyoxalase 2
LC-MS: liquid chromatography–mass spectrometry
