# Supplementary material for: PARK7 Catalyzes Stereospecific Detoxification of Methylglyoxal Consistent with Glyoxalase and Not Deglycase Function
Source: Biochemistry. 2023 Oct 26;62(21):3126–33. doi: 10.1021/acs.biochem.3c00325 (PMC10634309; doi:10.1021/acs.biochem.3c00325)
Supplement: Supplementary file 2 — bi3c00325_si_002.pdf [file bi3c00325_si_002.pdf]

Supporting Information:

PARK7 Catalyzes Stereospecific Detoxification of Methylglyoxal Consistent with Glyoxalase and not Deglycase Function

*John S. Coukos[1], Chris W. Lee[1], Kavya S. Pillai[1], Hardik Shah[2] & Raymond E. Moellering[1,2]**

[1]Department of Chemistry, The University of Chicago

929 E. 57th Street, Chicago, IL 60637, United States

[2]University of Chicago Medicine Comprehensive Cancer Center Metabolomics Platform, The University of Chicago

900 E. 57th Street, Chicago, IL 60637, United States

*e-mail correspondence to: rmoellering@uchicago.edu

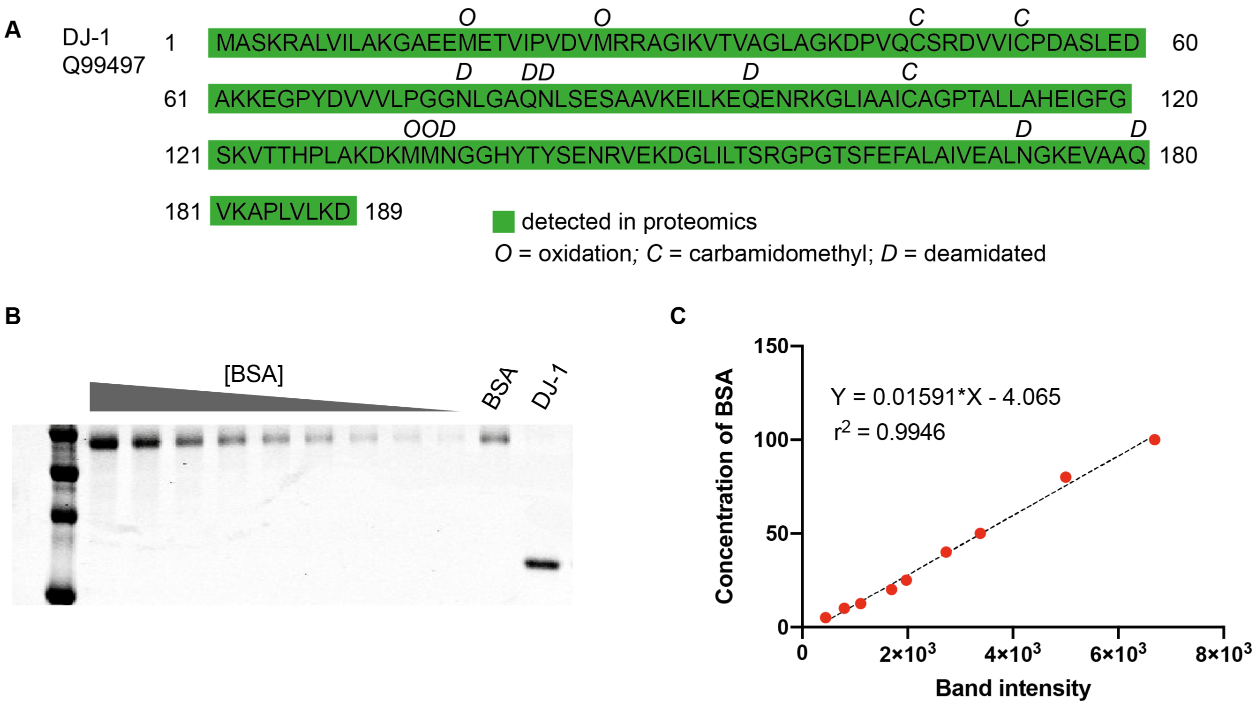

S1. Quantification of DJ-1 for enzymatic assays. **(A)** Sequence map of peptides detected by LC-MS/MS proteomic analysis of purified human DJ-1, which was used for experiments in this study. **(B-C)** Coomassie gel (B) of serial dilutions of BSA and quantification of the gel (C) used to estimate recombinant DJ-1 protein concentration.

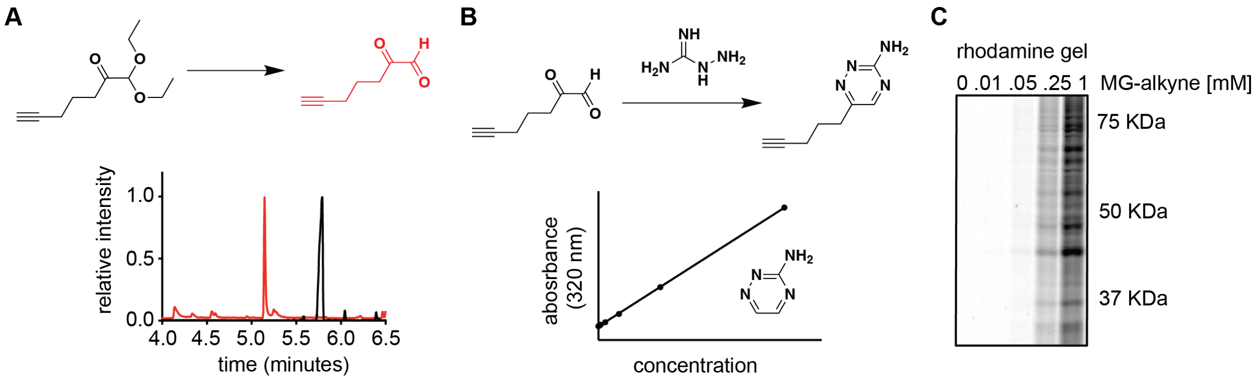

Supplemental Figure S2 Activation and evaluation of MG-alkyne probe **(A)** GC-MS traces of protected and deprotected MG-alkyne. **(B)** Schematic of the quantification method for MG-alkyne after deprotection. **(C)** Rhodamine gel of 2 mg/mL HEK293T lysate treated with the indicated concentrations of MG-alkyne for 2 hours at 37°C.

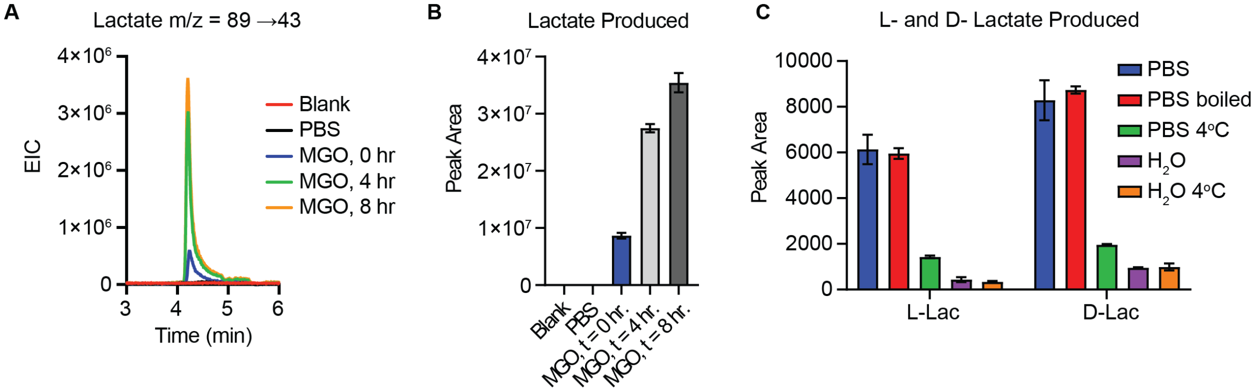

Supplemental Figure S3 Non-enzymatic production of lactate from methylglyoxal **(A)** Representative chromatograms of lactate formed from PBS treated with 1 mM MGO for 0, 4, and 8 hrs. at 37°C alongside vehicle and blank injection controls. Lactate is measured by MS/MS directly, without derivatization. **(B)** Relative quantification of lactate formed in (A). **(C)** Quantification of integrated peak area of L- and D- lactate formed from 1 mM MGO incubated in from PBS, freshly autoclaved PBS, or deionized water at either 4°C or 37°C. Data plotted in (B, C) are mean ± S.E.M from n = 4 independent biological replicates normalized across all conditions.

Materials/Experimental Details

All reagents were from Sigma-Aldrich, and all bulk solvents were from Thermo Fisher Scientific unless otherwise stated.

MG-alkyne synthesis.

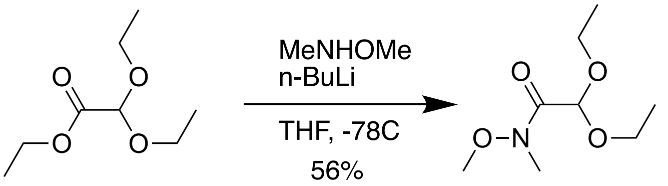

1

N,O-dimethylhydroxylamine (535 mg, 8.82 mmol) was dissolved in anhydrous THF (30 mL) under nitrogen and cooled to -78°C. n-Butyllithium (1.6 M in hexanes, 10.5 mL, 16.8 mmol) was added dropwise, then the solution was allowed to warm to r.t. and stir for 30 minutes. The reaction was again cooled to -78°C. Ethyl diethoxy-acetate (1.0 mL, 5.6 mmol) was added dropwise, and the reaction was warmed to r.t. and allowed to run for 3 hours. The reaction was quenched by the addition of saturated NH_4Cl solution (40 mL), extracted 3 times with ethyl ether, dried over $MgSO_4$, and concentrated in vacuo. The crude product **1** was purified by silica column (10% Et_2O in DCM), yielding a clear oil (600 mg, 56%): ^{1}H NMR ($CDCl_3$, 400 MHz) δ 5.30 (s, 1H), 3.75 (s, 3H), 3.72 (q, 4H, *J* = 5.0 Hz), 3.22 (s, 3H), 1.26 (t, 6H, *J* = 8.0 Hz).

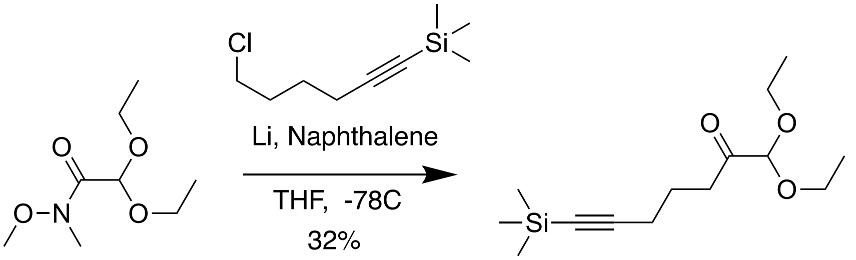

2

Napthalene (1.153 g, 9 mmol) and lithium metal (62.5 mg, 9 mmol) were added to anhydrous THF (30 mL) and sonicated at r.t. for 1 hour. The mixture was cooled to -78°C. (5-chloro-1-pentynyl)trimethylsilane (1.61 mL, 9 mmol) was added to anhydrous THF (15 mL) and then added to the reaction dropwise over the course of 30 minutes. The reaction was allowed to run for an additional 30 minutes and then the Weinreb amide starting material (575 mg, 3 mmol) was added dropwise and the reaction allowed to run for one hour. The reaction was quenched by the addition of saturated NH_4Cl solution (40 mL), extracted 3 times with ethyl ether, dried over $MgSO_4$, and concentrated in vacuo. The crude product **2** was purified by silica column (5% ethyl acetate in petroleum ether) yielding a clear oil (260 mg, 32%): ^{1}H NMR ($CDCl_3$, 400 MHz) δ 4.57 (s, 1H), 3.72-3.66 (m, 2H), 3.60-3.52 (m, 2H), 2.72 (t, 2H, *J* = 6.0), 2.27 (t, 2H, *J* = 8.0), 1.79 (tt, 2H, *J* = 8.0, 8.0), 1.26 (t, 6H, *J* = 8.0 Hz), 0.11 (s, 9H).

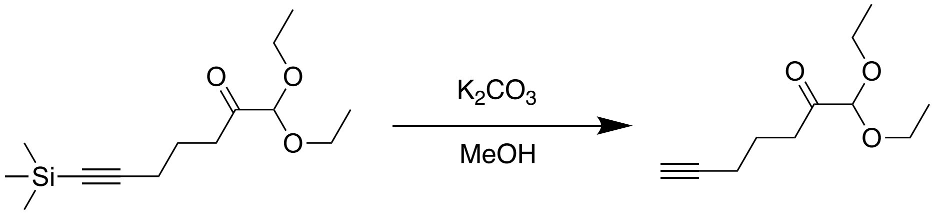

3

The starting material (125 mg, 0.46 mmol) and K_2CO_3 (380 mg, 2.76 mmol) were added to methanol (20 mL) and the reaction stirred at r.t. for 5 hours. The reaction was diluted with H_2O, extracted three times with DCM, washed once with H_2O, dried over $MgSO_4$, and concentrated in

vacuo. The crude product **3** was purified by silica column (5% acetone in pentane) yielding a clear oil (90 mg, 98%): ^{1}H NMR ($CDCl_3$, 400 MHz) δ 4.57 (s, 1H), 3.74-3.66 (m, 2H), 3.60-3.53 (m, 2H), 2.74 (t, 2H, *J* = 6.0), 2.24 (dt, 2H, *J* = 4.0,6.0), 1.81 (tt, 2H, *J* = 8.0, 8.0), 1.25 (t, 6H, *J* = 8.0 Hz).

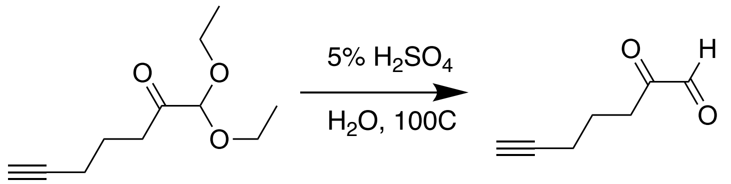

"MG-alkyne"

The starting material (34 mg, 0.17 mmol) was added to 1 mL of 5% H_2SO_4 solution and heated to a reflux for 1 hour. 10% NaOH solution (0.5 mL) was added to partially neutralize the reaction, then the reaction mixture was diluted with 50 mM sodium phosphate buffer pH 7.4. The probe solution was used as crude mixture without purification. Complete deprotection was confirmed by GC-MS (Figure S2, theoretical m/z 125.05, observed 125.0). The deprotected product MG-alkyne was quantified as described below.

Methylglyoxal synthesis and distillation. High purity MGO was prepared by acidic hydrolysis of MG-1,1-dimethylacetal followed by fractional distillation[53]. Briefly, 6 mL of MG-1,1-dimethylacetal was added to 100 mL of 2.5% (v/v) sulfuric acid and refluxed for 1 hour. The product was purified by fractional distillation under reduced pressure. The first fraction collected was discarded due to methanol impurity.

MGO and MG-alkyne quantification. MGO and MG-alkyne concentrations were quantified by colorimetric assay[53]. Briefly, samples were diluted to below 2 mM concentration as needed and reacted with equal volume of 40 mM aminoguanidine in phosphate buffer for 5-6 hours at 37°C. Absorbance was measured at 320 nm and compared to a calibration curve generated by serial dilutions of 3-amino-1,2,4-triazine in phosphate buffer.
MGO fractions were then diluted to 50 mM stock solutions using phosphate buffer and pH was confirmed to be 7.4. MGO stocks were stored at -80°C until use.
Deprotected MG-alkyne was diluted 25 mM stock solutions using phosphate buffer and pH was confirmed to be 7.4. MG-alkyne stocks were stored at -80°C until use.

Cell culture. HEK293T cells were purchased from ATCC and were propagated in RPMI 1640 with 2 mM glutamine supplemented with 10% fetal bovine serum and 1% penicillin/streptomycin (Gibco).

Plasmids and cloning. The pDONR221-DJ-1 was generated by cloning a gene block purchased from Integrated DNA Technologies (IDT) containing the human *PARK7* (UniProt: Q99497) open reading frame into the pDONR221 plasmid. The pHAGE-CMV-C-Flag-HA-DJ-1 plasmid was generated via Gateway LR clonase (Thermo Fisher Scientific) reaction with the pDONR221-DJ-1 and pHAGE-CMV-C-Flag-HA-Puro plasmids.

Generation of stable cell lines. HEK-Flag-DJ-1 cells were generated by transducing HEK293T cells with the pHAGE-CMV-C-Flag-HA-DJ-1 plasmid. Briefly, lentiviruses were generated in HEK293T cells by transient transfection of pHAGE-CMV-C-Flag-HA-DJ-1 plasmid with pSPAX2 and pMD2.G packaging vectors (Addgene plasmids #11260 and #12259) using lipofectamine 2000 (Thermo Fisher Scientific). Viral supernatants were collected after 48 hours of expression, passed through a 45-μm syringe filter, and supplemented with 8 μg/mL of polybrene

(hexadimethrine bromide) before exposure to target cells. Selection was performed with 2 μg/mL puromycin.

Western blotting. Cells were washed with PBS buffer, collected by scraping in PBS buffer, resuspended in PBS buffer containing EDTA-free complete protease inhibitors (Roche), and sonicated (Fisher Scientific FB-505) for 15 seconds (30% amplitude, 1 second on, 1 second off). Insoluble debris was cleared by a 15-minute centrifugation at 16,000g and 4°C. Protein concentrations were normalized via BCA assay. The supernatant was diluted into 4x Laemmli buffer containing 100 mM βME. Samples were prepared for SDS-PAGE by heating to 95°C for 5 min, cooled to room temperature, resolved on a 10% or 15% SDS-PAGE gel depending on the proteins of interest, and transferred onto nitrocellulose membrane by standard western blotting methods. Membranes were blocked in 2% BSA in TBS containing 0.1% Tween-20 (TBST) and probed with primary and secondary antibodies. Antibodies used in this study include: anti-Flag-M2 (1:3000, F1804, Sigma Aldrich) and secondary donkey anti-mouse 680 (Licor,1:10,000). Blots were imaged on a Licor infrared scanner.

Rhodamine labeling and imaging. For in-gel fluorescence readout, 50 μL of MG-alkyne-treated sample was reacted with 1 μL of 50 mM $CuSO_4$, 1 μL of 50 mM TCEP-HCl, 3 μL of 1.67 mM TBTA in 4:1 t-butanol/DMSO, and 1 μL of rhodamine-azide (1 mM stock in DMSO) at RT for 1 hour. The samples were diluted into 4x Laemmli buffer containing 100 mM βME. Samples were prepared for SDS-PAGE by heating to 95°C for 5 min, cooled to RT, resolved on a 10% SDS-PAGE gel, and imaged on a BioRad ChemiDoc MP Imager.

MG-alkyne in vitro dose response and competition samples. HEK293T cells were washed with PBS buffer, collected by scraping in PBS buffer, resuspended in PBS buffer containing EDTA-free complete protease inhibitors (Roche), and sonicated (Fisher Scientific FB-505) for 15 seconds (30% amplitude, 1 second on, 1 second off). Insoluble debris was cleared by a 15-minute centrifugation at 16,000g and 4°C. Protein concentrations were determined by Bradford assay, and samples were diluted to 2 mg/mL with PBS buffer containing EDTA-free complete protease inhibitors. Samples were treated for 2 hours with indicated concentrations of MGO at 37°C followed by 2-hour treatment with indicated concentrations of MG-alkyne at 37°C. Samples were subsequently rhodamine labeled for in gel-fluorescence imaging.

DJ-1 Flag purification. HEK-Flag-DJ-1 cells were grown to confluence in a 10 cm dish then washed with PBS buffer, collected by scraping in PBS buffer, resuspended in PBS buffer containing EDTA-free complete protease inhibitors (Roche), and sonicated (Fisher Scientific FB-505) for 15 seconds (30% amplitude, 1 second on, 1 second off). Insoluble debris was cleared by a 15-minute centrifugation at 16,000g and 4°C. Lysates were incubated overnight at 4°C with pre-washed M2 anti-Flag affinity resin (Sigma Aldrich). The Flag resin was washed 5 times with 1 mL of PBS and then the Flag-DJ-1 protein was eluted from the resin by incubating it in 300 μL of PBS with 100 μg/mL 3xFlag peptide at r.t. for 1 hour. The resin was pelleted by centrifugation at 4000g for 2 minutes, and the supernatant containing the Flag-DJ-1 was kept for further experiments. Protein concentration was determined via coomassie stain using a calibration curve with serial dilutions of BSA.

DJ-1 enzymatic assay. 1 μg of recombinant DJ-1, BSA, or vehicle were added to 50 μL of 1 mM MGO or MG-alkyne in PBS and incubated for 24 hours at 37°C. Final MGO and MG-alkyne concentrations were determined as described above. For LC-MS/MS analysis of MG-alkyne enzymatic product, samples were acidified with 0.2% TFA, centrifuged at 16,000g for 3 minutes, and the supernatant kept. Data presented are representative of three independent experiments.

L- and D-Lactate enantiomer quantification. 1 µg of recombinant DJ-1, BSA, or vehicle were added to 50 µL of 1 mM MGO in PBS and incubated at 37°C for 24 hours or for the indicated time points in the kinetic assay. For non-enzymatic conversion assays, 1 mM MGO was added to 50 µL of commercial cell culture grade PBS (Corning, 21-031-CV) or Milli-Q Ultra-pure water (Sigma) that was either used directly or first boiled at 100°C for 30 mins, de-gassed for 15 mins, and then cooled to room temp. Samples were then incubated at 37°C or kept on ice for 8 hours.

Following incubations, samples were then derivatized with (+)-*O,O'*-Diacetyl-L-tartaric anhydride (DATAN) and then analyzed by LC-MS/MS as previously described[55]. Briefly, samples and L- and D- lactate standards were dried under N_2 stream and then resuspended in 100 µL of 50 mg/mL DATAN in 4:1 vol/vol DCM/acetic acid solution and heated at 75°C for 30 min. Samples were then again dried under N_2 stream and then resuspended in H_2O for LC-MS/MS analysis. Targeted MS/MS analyses were performed on an Agilent triple quadrupole LC-MS/MS instrument (Agilent Technologies 6460 QQQ) with 1290 UHPLC and 1260 nanoLC-Chip set to negative ion mode with a mass window of 50 to 1000 m/z. The capillary voltage was set to 4.0 kV. The drying gas temperature was 300°C, flow rate = 5 L/min, and nebulizer pressure = 45 psi. The mass spectrometer was run in MRM mode with precursor ion of 305, product ion of 89.1, MS1 resolution set to wide, MS2 resolution set to unit, dwell time of 100 ms, a fragmentor voltage of 100V, and collision energy of 14V. Chromatography was performed with an Acquity UPLC HSS T3 analytical column (2.1x100 mm, 1.8 µm, Waters) held to 40°C. Mobile phase A was composed of 125 mg/L ammonium formate in H_2O adjusted to pH 3.5 with formic acid, and mobile phase B was composed of methanol. The instrument was run at 0.3 mL/min with the following gradient: 3% Buffer B (0-5 min); 3-80% Buffer B (5-5.5 min); 80% Buffer B (5.5-8 min); 80-5% Buffer B (8-8.5 min); 5% Buffer B (8.5-18.5 min). Relative abundance was quantified by integrated peak area.

Non-enzymatically produced lactate quantification by LC-MS/MS without DATAN derivatization. 50 µL of 1 mM MGO in PBS was incubated for 0-8 hours at 37°C. The reaction was then quenched in 80/20 methanol/water before being dried under N_2 stream. The dried down samples were re-suspended in 60 µL of 60/40 acetonitrile/water, sonicated for 3 minutes, vortexed for 5 minutes at 4°C using an Eppendorf ThermoMixer, centrifuged at 20,000g for 15 minutes and the supernatant was transferred in LC-MS vial for analysis.
Metabolite separation was performed using a Thermo Scientific Vanquish Horizon UHPLC system and XBridge BEH amide 2.5 µm (2.1x150 mm Column XP, Waters Corporation, MA) under acidic conditions. Mobile phase A (MPA) was 90/5/5 water/acetonitrile/methanol, 20 mM ammonium acetate, 0.2% acetic acid and mobile phase B (MPB) was 90/10 acetonitrile/water, 10 mM ammonium acetate, 0.2% acetic acid. The column temperature was 40°C, flow rate was 0.3 mL/min, and injection volume was 5 µL. The chromatographic gradient was: 0min: 95% B, 9min: 70% B, 9.75min: 40% B, 12min: 40% B, 13min: 30% B, 14min: 30%B, 14.1min: 10% B, 17min: 10% B, 17.5min: 95% B, 22min: 95% B. An Orbitrap IQ-X Tribrid mass spectrometer (Thermo Scientific) with a H-ESI probe operating in negative polarity was utilized for MS detection using the following parameters: Acquisition range of 70-1000 m/z at 60K resolution, spray voltage: 2800V, sheath gas flow: 40, aux gas flow: 7, sweep gas flow: 1, AGC target: 100, maximum injection time: 118 ms, capillary temperature: 250°C, RF level: 60 and aux gas heater temperature: 350°C. Additionally, Selected Ion Monitoring (SIM) was utilized to collect high resolution fragmentation for lactate (m/z 89.0244) using the following parameters: isolation mode: quadrupole, isolation window (m/z): 0.8, normalized HCD collision energy: 30%, and

detector type: orbitrap at 30K resolution. Data acquisition was done using the Xcalibur software (Thermo Scientific) and data analysis was performed using Tracefinder 5.1 software (Thermo Scientific). Lactate peak was confirmed by matching the retention time and MS/MS fragmentation (m/z 43.0185 fragment ion) to an in-house database generated using a reference standard.

LC-MS/MS analysis of DJ-1 MG-alkyne product. Samples were analyzed on an Agilent 6540 Q-TOF MS-MS with 1290 UHPLC and 1260 nanoLC-Chip set to positive ion mode with a mass window of 50 to 1000 m/z. The capillary voltage was set to 3.5 kV. The drying gas temperature was 300°C, the drying gas flow rate was 8 L/min, and the nebulizer pressure was 35 psi. The fragmentor voltage was set to 150 V. Chromatography was performed with a Phenomenex Gemini C18 column (50 x 4.6 mm, 5 μM) at a flow rate of 0.4 mL/min. Mobile phase A was composed of H_2O supplemented with 0.1% TFA, and mobile phase B was composed CH_3CN supplemented with 0.1% TFA. The instrument was run at 0.4 mL/min with the following gradient: 0% Buffer B (0-2 min); 0-60% Buffer B (2-7 min); 60-100% Buffer B (7-8 min); 100% Buffer B (8-10 min); 100-0% Buffer B (10-11 min); 0% Buffer B (11-13 min). Injection volume was 5 µL for all samples. Data presented are representative of three independent experiments.

DJ-1 glycation assay with BSA. 1 µg of DJ-1 or vehicle were added to 100 µL of 1 mg/mL BSA and either 100 or 300 µM MG-alkyne. The reactions were incubated for 24 hours at 37°C and then labeled with rhodamine azide for gel-based analysis as described above. Densitometry measurements were performed with ImageJ software.

Membrane assay. 1.1 mL of 1.36 mg/mL BSA in PBS was added to 1.7 mL microcentrifuge tubes. For the membrane containing conditions, a 0.5 mL Slide-A-Lyzer MINI dialysis device with 3.5 KDa cutoff (Thermo Fisher Scientific) was pre-wet and then added to the tube and rotated tight to ensure there were no air bubbles present between the solution and the dialysis membrane. 0.4 mL of PBS with or without 15 µg recombinant DJ-1 was added to each reaction followed by 6 µL of 25 mM MG-alkyne. The reactions were incubated for 24 hours at 37°C, samples containing 50 µg of BSA from each reaction were taken, and volumes were equalized across samples to 50 µL via the addition of PBS as needed. Samples were labeled with rhodamine azide for gel-based analysis as described above. Densitometry measurements were performed with ImageJ software.

GC-MS analysis of MG-alkyne deprotection. Diethyl acetal-protected MG-alkyne and hydrolyzed MG-alkyne product were analyzed using an Agilent 5977A MSD with installed EI/CI ion source connected to an Agilent 7890B GC system with an Agilent 7693 autosampler. Samples were run using a 13-minute temperature gradient where temperature was held at 50°C for 1 minute, followed by a linear increase to 250°C over 5 minutes, and then held at 250°C for 7 minutes an acquisition delay of 3.20 minutes was used to avoid the solvent front. Samples were analyzed in PCI mode with a scan range of 100 to 500 Da with a 0.1 m/z step size and 3.6 scans/second collected.
